# Simulated Sunlight Selectively Modifies Maillard Reaction Products in a Wide Array of Chemical Reactions

**DOI:** 10.1002/chem.201902804

**Published:** 2019-09-13

**Authors:** Daniel Hemmler, Michael Gonsior, Leanne C. Powers, James W. Marshall, Michael Rychlik, Andrew J. Taylor, Philippe Schmitt‐Kopplin

**Affiliations:** ^1^ Comprehensive Foodomics Platform, Analytical Food Chemistry Technical University Munich Maximus-von-Imhof-Forum 2 85354 Freising Germany; ^2^ Research Unit Analytical BioGeoChemistry (BGC) Helmholtz Zentrum München Ingolstädter Landstrasse 1 85764 Neuherberg Germany; ^3^ University of Maryland Center for Environmental Science Chesapeake Biological Laboratory Solomons USA; ^4^ The Waltham Centre for Pet Nutrition Mars Petcare (UK) Waltham-on-the-Wolds Leicestershire LE14 4RT UK

**Keywords:** advanced glycation, Maillard reaction, photochemistry, photooxidation, reactive oxygen species

## Abstract

The photochemical transformation of Maillard reaction products (MRPs) under simulated sunlight into mostly unexplored photoproducts is reported herein. Non‐enzymatic glycation of amino acids leads to a heterogeneous class of intermediates with extreme chemical diversity, which is of particular relevance in processed and stored food products as well as in diabetic and age‐related protein damage. Here, three amino acids (lysine, arginine, and histidine) were reacted with ribose at 100 °C in water for ten hours. Exposing these model systems to simulated sunlight led to a fast decay of MRPs. The photodegradation of MRPs and the formation of new compounds have been studied by fluorescence spectroscopy and nontargeted (ultra)high‐resolution mass spectrometry. Photoreactions showed strong selectivity towards the degradation of electron‐rich aromatic heterocycles, such as pyrroles and pyrimidines. The data show that oxidative cleavage mechanisms dominate the formation of photoproducts. The photochemical transformations differed fundamentally from “traditional” thermal Maillard reactions and indicated a high amino acid specificity.

## Graphical abstract

**Here comes the sun**: Sunlight selectively decomposes Maillard reaction products into hundreds of new compounds. Within the great pool of thermally formed Maillard reaction products, especially aromatic heterocycles are good targets for a rapid and photon‐triggered breakdown into smaller and highly oxidized structures.

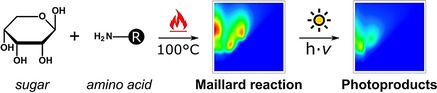

## Introduction

Non‐enzymatic browning reactions have been of great interest in food science and health. In food products, reactions between amino acids and carbonyl moieties (Maillard reaction, MR) are the main contributors to flavor and color formation.^1^, ^2^ Under physiological conditions, non‐enzymatic glycation leads to irreversible protein damage (advanced glycation endproducts, AGEs), associated with a wide range of diseases.^3^ Non‐enzymatic browning leads to a heterogeneous class of compounds including chromophores and fluorophores that absorb and emit in the ultraviolet (UV) and visible (Vis) spectral range. Aromatic and often heterocyclic colored compounds are formed mainly in the final phase of the MR by a series of condensation reactions, many of which are only partly understood.^4^

During their shelf life, food products are often unavoidably exposed to sunlight. In a similar way, AGEs, for example, in eye lenses or skin, continuously experience solar exposure. Chromophores formed as part of the advanced glycation have been suggested as possible photosensitizers producing reactive oxygen species (ROS), which lead to age‐related protein photodamage. Major targets for photo‐oxidation reactions in proteins are aromatic amino acids (tryptophan, Trp, tyrosine, Tyr, and phenylalanine, Phe) as well as histidine (His), cysteine (Cys), and methionine (Met) residues.^5^ The amino acids lysine and arginine, which are of greatest relevance in non‐enzymatic glycation reactions on proteins, do not show significant absorption at wavelengths >230 nm^6^ and photooxidation on these amino acids has only been observed at high pH values for their unprotonated species.^7^ Increased levels of AGE photosensitizers have been found in aged and diabetic lenses^8^ as well as on long‐lived skin proteins.^9^ It is well accepted that the predominating mechanism involves the AGE sensitized formation of ROS, such as singlet oxygen (^1^O_2_), superoxide anion radicals (^⋅^O_2_^−^), and hydroxyl radicals (^⋅^OH).^10^, ^11^ Furthermore, Wondrak and co‐workers showed that AGEs can act as photosensitizers to DNA damage. In addition to reactions of ROS, they also proposed other photosensitization reactions to be involved in phototoxicity mechanisms.^12^

Given the chemical nature of reducing sugars, the initial condensation with amine compounds and subsequent downstream reactions lead to a wide range of compounds with carbonyl functional groups, including dicarbonyl moieties and α‐hydroxy ketones.^2^, ^13^ When irradiated with UV‐B light, carbonyl functional groups can form acyl radicals in aqueous solutions, predominantly by Norrish Type‐I photofragmentation reactions.^14^, ^15^, ^16^ The α‐cleavage and subsequent decarbonylation is about an order of magnitude faster for α‐hydroxy ketones than for their alkyl counterparts.^15^ Although the nature of the compound classes formed during the MR suggests a strong photochemical reactivity, to date, the effect of solar radiation on the direct chemical alteration of MRPs is only partially explored. Bohart and Carson were the first to report discoloration in glucose–glycine Maillard model systems when they were exposed to illumination under oxygen in the laboratory.^17^ Later, Kessel and co‐workers showed that UVA radiation readily degrades purified argpyrimidine.^18^

Herein, we report the photochemical effects on MRPs using a comprehensive non‐targeted analysis. More precisely, we exposed reaction products, initially formed by heating different amino acid–ribose mixtures at 100 °C for ten hours, to a simulated solar spectrum. Chemical changes were analyzed by optical spectroscopy and (ultra)high‐resolution mass spectrometry. We focus on three different amino acids (lysine, arginine, and histidine), the MRPs of which provide abundant chromophores. Although lysine and arginine are the main contributors to non‐enzymatic glycation reactions in foods and on proteins under physiological conditions, histidine is among the major protein residues for ROS‐driven cellular photodamage. Ribose has been chosen because of its high reactivity, readily leading to a large number of MRPs upon thermal processing.

## Results and Discussion

### Effect of simulated solar irradiation on absorption and fluorescence properties of Maillard reaction products

#### Excitation–emission matrix measurements of MRPs and during photodegradation experiments

**Figure 1:**
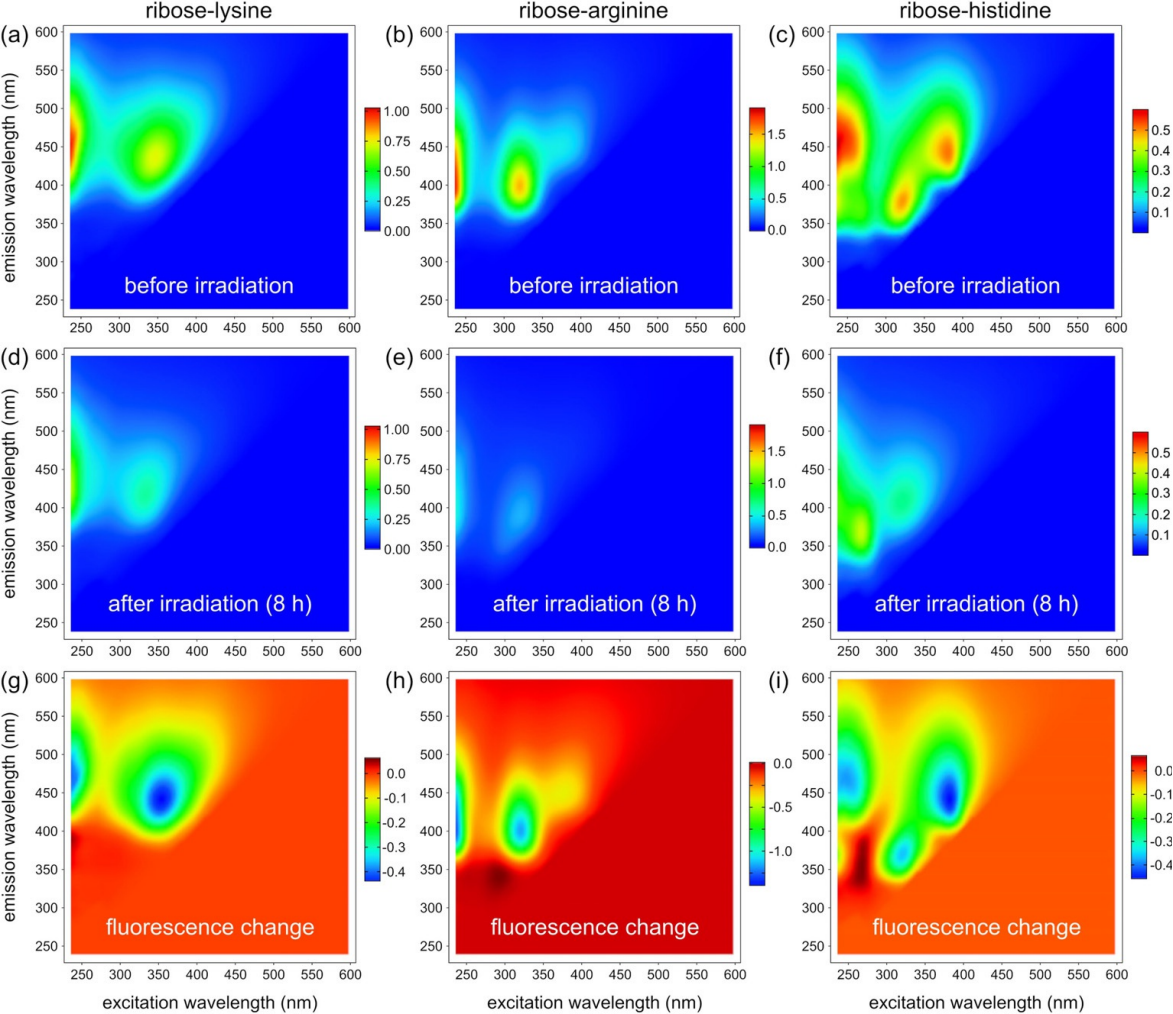
Photolytic degradation of MRPs in three model systems (ribose–lysine, –arginine, and –histidine) heated for ten hours at 100 °C. Excitation–emission matrices retrieved from diluted model systems (1:800 *v*/*v* in H_2_O) (a–c) before irradiation and (d–f) after solar irradiation for 8 h. (g–i) Changes in fluorescence intensity after an irradiation time of 8 h compared to nonradiated samples. All fluorescence intensity values are expressed in quinine sulfate units (ppm).

After an induction period, heating reducing sugars in the presence of amino acids leads to the formation of chromophores and fluorophores.^19^ Among the proteinogenic amino acids, especially those with basic functional side chains, significant color formation occurs in unbuffered solutions whereas the amino acids themselves do not disturb fluorescence detection.^20^ After heating three different model systems (ribose–lysine, ribose–arginine, and ribose–histidine) for ten hours at 100 °C excitation–emission‐matrices (EEM) were constructed from fluorescence measurements (Figure 1 a–c). Fluorescence intensities and excitation/emission wavelengths strongly depended on the amino acid precursor. Interestingly, ribose–histidine showed the most complex fluorescence behavior with at least two major emitting regions indicating multiple chemical structures and moieties participating in the overall fluorescence behavior (Figure 1 c). In the ribose–lysine and –arginine model systems we observed dominating fluorescence peaks with emission maxima at 440 nm (excitation: ≤245 and 350 nm; Figure 1 a) and 400 nm (excitation: ≤245 and 320 nm; Figure 1 b), respectively. Only a few fluorescent MRPs have been previously fully characterized. Most of them were isolated from model systems containing the amino acids lysine and arginine.^21^ Many of the studied fluorescent MRPs are involved in the crosslinking of proteins and are used as important markers in the formation of AGEs. Pentodilysine (LM‐1), a fluorescent molecule cross‐linking lysine residues, has excitation/emission wavelengths that would match the dominating fluorescence peak in the ribose–lysine model system (Figure 1 a).^22^, ^23^ In a similar way, argpyrimidine shows excitation/emission corresponding to the major peak found in the ribose–arginine model system (Figure 1 b).^24^ Although pentodilysine can be formed directly from ribose and lysine, argpyrimidine is formed from arginine and methylglyoxal.^23^, ^24^ Fluorophores formed in the Maillard reaction by other amino acids than lysine and arginine have received only minor attention^21^ even though some studies support that fluorescent MRPs may be related to the formation of brown pigments.^20^

**Figure 2:**
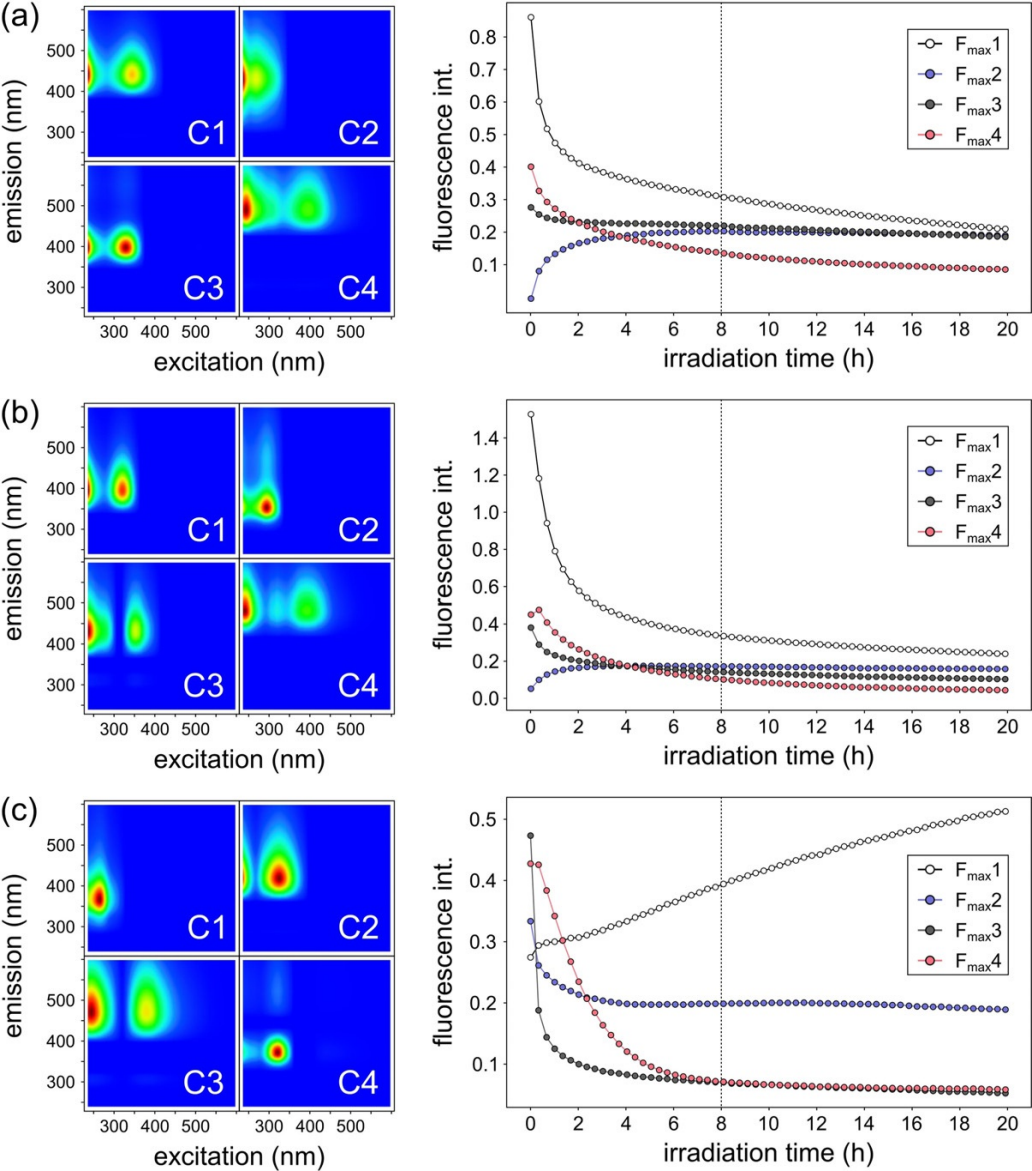
Four component EEM‐PARAFAC models obtained from EEM measurements. (a) Ribose–lysine, (b) ribose–arginine, and (c) ribose–histidine model systems. All model systems were irradiated for 20 h. EEM spectra were recorded every 20 min.

The same model systems (ribose–lysine, –arginine, and –histidine) were then exposed to solar irradiation to initiate photodecomposition (e.g. photobleaching and phototransformations). Upon solar irradiation, we observed a fast decay in fluorescence intensities in all model systems (Figure 1 d–i). In addition to the decrease in fluorescence, however, the EEM maps in Figure 1 g–i also indicate formation of new fluorescent compounds, which were formed during the photolysis or whose fluorescence has been quenched by other compounds prior to irradiation. Parallel factor analysis (PARAFAC) was used to decompose fluorescence spectra into distinct statistical components.^25^ For all three model systems, four‐component PARAFAC models were developed and split‐half validated. In addition to the above‐mentioned characteristic spectral regions, the PARAFAC models give evidence for the existence of additional fluorophores, even though with lower quantum yields (Figure 2 and Table 1). For all model systems, three components were retrieved which showed a decrease in fluorescence and one fluorescent component which increased over time, respectively. These components (Lys: C2, Arg: C2, His: C1) show very different excitation/emission positions and different Stoke shifts between the model systems indicating different chemical structures and fluorescent moieties, respectively. In the ribose–lysine model system (Figure 2 a), the increase of C2 showed remarkable correlation to the decreasing components. Hence, C2 may be formed by photochemical reactions from other fluorophores or the other fluorophores may have quenched the fluorescence of C2 before irradiation. A similar correlation could be found for C2 and C3/C4 in the ribose–arginine reaction system (Figure 2 b). Very similar fluorescence peaks further indicate the possibility of similar substructures (fluorophoric groups) between C4 of the ribose–lysine and C4 of the ribose–arginine systems (Figure 2 a–b). In general, the photokinetics derived from the PARAFAC components are very similar between the lysine and arginine reaction systems (Figure 2 a–b). In contrast, fluorophores degraded and potentially formed in the ribose–histidine model system behaved differently (Figure 2 c). For example, the increasing component (C1) showed a nearly linear increase in fluorescence over the entire irradiation period indicating zero‐order photochemical synthesis.

**Table 1:** Fluorescence local maxima obtained by EEM‐PARAFAC analysis. All values are given in nm. Ex: excitation, Em: emission

Component	Lysine		Arginine		Histidine	
	Ex	Em	Ex	Em	Ex	Em
C1	350	440	320	400	265	365
C2	270	430	295	350	330	420
C3	330	395	355	430	245 380	470 470
C4	240 395	485 485	245 395	480 480	325	370

#### Absorbance measurements

**Figure 3:**
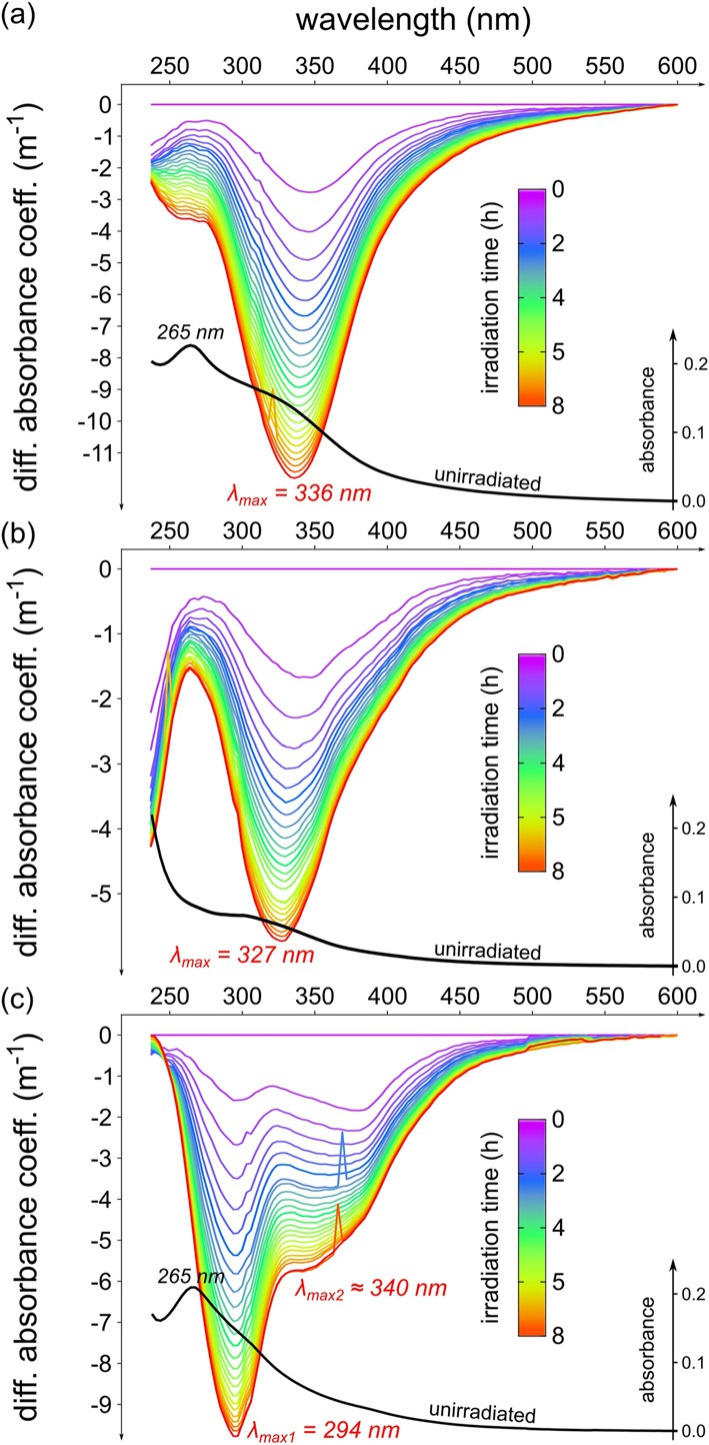
Changes in the absorption spectra upon irradiation. Differential absorbance spectra of (a) ribose–lysine, (b) ribose–arginine, and (c) ribose–histidine model systems irradiated for eight hours. UV/Vis spectra were recorded every 20 minutes simultaneously with EEMs presented above. Embedded black curves represent UV/Vis absorption spectra of unirradiated model systems (10 h, 100 °C), respectively.

After heating the ribose–amino acid mixtures for ten hours at 100 °C, we found maximum absorbance at 265 nm in the spectra of the ribose–lysine and –histidine mixture (Figure 3 a and Figure 3 c). The arginine reaction system showed maximum absorbance <240 nm but indicated a second maximum at about 300 nm (Figure 3 b). Irradiation of the samples led to an exponential decrease in absorbance with maximum decrease found at 336, 327, and 294 nm for the ribose–lysine, –arginine, and –histidine model system, respectively (Figure 3). The discrete maxima indicate a selectivity of photochemical reactions rather than random degradation of all chromophores absorbing in the irradiated energy range. A redshifted shoulder in Figure 3 c (ribose–histidine model system) indicated an underlying curve with a second maximum decrease in absorbance at approximately 340 nm, which was in the range found for the ribose–lysine and ribose–arginine model systems (Figure 3 a–b), and could represent degradation reactions on chromophoric groups, similar to those in the lysine and arginine systems. The relative degradation rates of the two maxima found at 294  and 340 nm in the ribose–histidine model system were different. Especially at the beginning of the irradiation process, degradation rates at 340 nm were faster than for chromophores showing greatest changes at 294 nm (Figure 3 c). This suggests at least two different groups of chromophores and potentially different degradation mechanisms.

### Holistic characterization of photosensitive MRPs

We additionally irradiated larger amounts of sample in a suntester solar‐simulation system equipped with a xenon arc lamp, also simulating the solar spectrum. Irradiation was performed for four and eight hours in the course of which the temperature was maintained at 25 °C. Control samples, which were protected from light exposure, were also placed in the suntester system for four and eight hours. Subsequently, we analyzed the samples by direct‐infusion Fourier transform ion cyclotron resonance mass spectrometry (FT‐ICR‐MS) and tandem‐column LC‐MS/MS, which combines hydrophilic interaction chromatography (HILIC) and reversed‐phase (RP) liquid chromatography (LC) in a single chromatographic run.^26^ Direct‐infusion FT‐ICR‐MS allows a highly sensitive and holistic nontargeted screening of complex samples on the level of accurate molecular formulae.^27^ Principal component analysis (PCA) of the obtained FT‐ICR‐MS raw data could clearly separate irradiated model systems from controls on PC1 (exemplarily shown for the ribose–histidine model in Figure S1, Supporting Information). Samples irradiated for four hours could also be distinguished from samples exposed to sunlight for eight hours by PC2. Moreover, we could not observe a difference between the control samples kept for four and eight hours in the suntester (while protected from light exposure) and freshly prepared model systems. This indicates that no significant thermal or time effects on the formation of new MRPs occurred during irradiation experiments.

#### Photochemical degradation of MRPs

**Figure 4:**
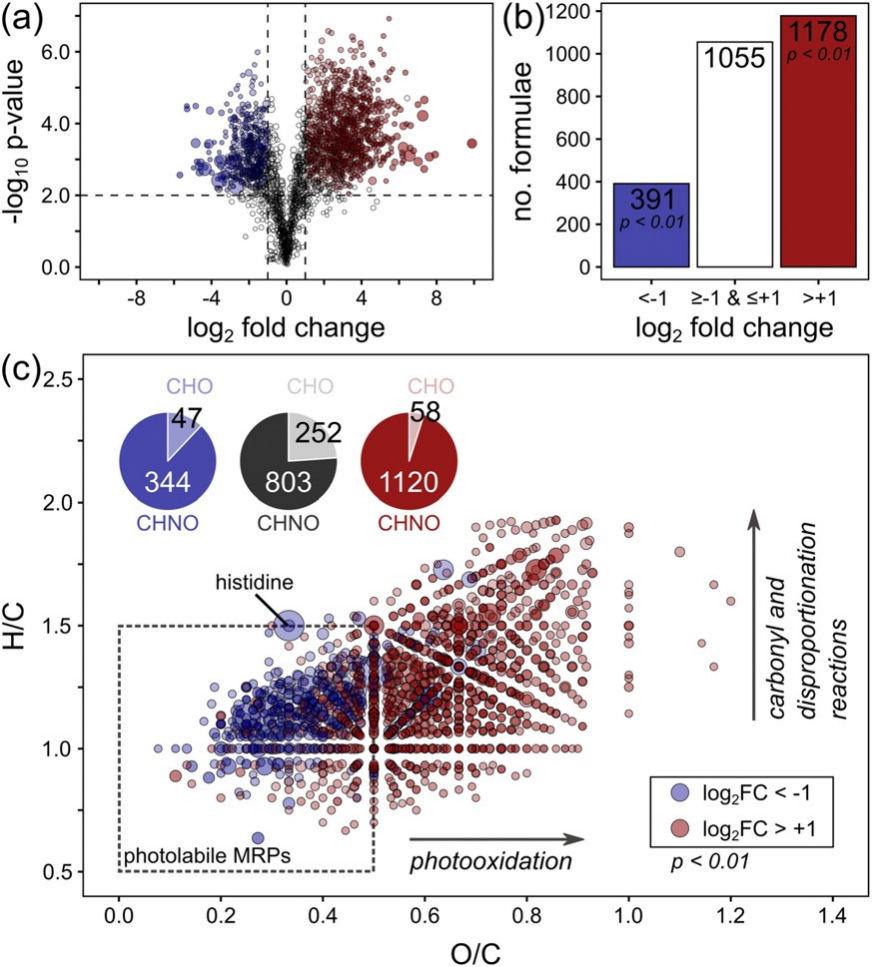
Effect of solar irradiation on elemental compositions of ribose–histidine MRPs. Model systems were irradiated for eight hours and compared to unirradiated control samples. Irradiation experiments were performed in duplicate. Each sample then was analyzed by FT‐ICR‐MS in triplicate injections (*N*=2×3). Peak intensities of all features found in irradiated samples were compared to the same features in the unirradiated control samples by Student's t‐Test (*n*=3): Features, which showed a significant decrease in peak intensities in both independent irradiation experiments are colored in blue. Features, which showed a significant increase or were newly formed upon irradiation are highlighted in red, respectively. (a) Volcano plot. (b) Number of molecular formulae showing significant changes in peak intensities. (c) Van Krevelen diagram^28^ of all significantly affected molecular formulae. Pie charts illustrate the reduced occurrence of nitrogen‐free (CHO) MRPs in photochemical reactions. Black pie chart represents elemental compositions, which did not show a significant change in peak intensities upon irradiation.

In total, we could detect 1446, 1945, and 2066 monoisotopic signals in unirradiated ribose–histidine, –lysine, and –arginine model systems, respectively, using FT‐ICR‐MS. Upon solar irradiation, we observed the largest changes in the ribose–histidine model system. Here, 391 elemental compositions (20 %) showed a significant decrease in peak intensities after an irradiation time of eight hours (p<0.01 and log_2_FC<−1 in both replicate experiments, Figure 4). By comparison, in ribose–lysine and –arginine model systems irradiation led to 42 (2.2 %) and 88 (4.3 %) elemental compositions, which showed a significant decrease in signal intensities (Figures S2–S3, Supporting Information).

#### Photooxidation of MRPs by singlet oxygen

Histidine residues are well known to easily undergo photooxidation reactions.^5^, ^29^, ^30^ However, direct light absorption by the imidazole function is not the major mechanism.^30^ In oxygen‐rich atmospheres, histidine photooxidation mainly occurs through Type‐II photo reactions.^31^ In a Type‐II reaction, the energy absorbed by a sensitizer is transferred onto ground‐state molecular oxygen to produce singlet oxygen (^1^O_2_) and to a lesser extent other ROS.^11^ Singlet oxygen then may form unstable endoperoxide intermediates on imidazole residues, which further decompose into a complex mixture of as yet mostly unknown products.^32^, ^33^ Among the proteinogenic amino acids, only Trp, Tyr, Phe, His, Cys, and Met show noteworthy rate constants in the reaction with ^1^O_2_ with the highest values found for histidine oxidation,^34^ which might explain the greater number of photolabile MRPs in the ribose–histidine model compared to the two other model systems investigated. Nevertheless, formation of ^1^O_2_, and subsequent reactions of MRPs with ^1^O_2_ may also play a role in the ribose–lysine and –arginine model systems, particularly due to photosensitizers that can be produced in the course of the MR. We could detect significantly elevated levels of urea and asparagine in the irradiated histidine model systems (Figure S4, Supporting Information), which are formed in the decomposition of histidine through ^1^O_2_.^33^, ^35^ Assuming that urea and asparagine are exclusively formed from photooxidation reactions, quantification (by LC‐MS) of the formed urea and asparagine, suggested up to 0.06–0.09 % and 0.002 % of the initial histidine amount being transferred into urea and asparagine, respectively (Figure S4). Although urea can also be formed by α‐NH_2_‐substituted histidines, asparagine can only be formed by degradation of the free amino acid.^33^

**Figure 5:**
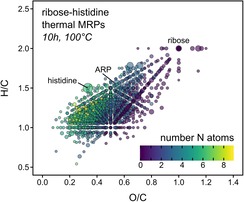
Van Krevelen diagram^28^ of all molecular formulae reproducibly found in two independent replicate experiments (each analyzed in triplicate) after heating a ribose–histidine model system for ten hours at 100 °C. Color indicates the number of nitrogen atoms in the formulae. Scaling is relative to the average peak intensity recorded by FT‐ICR‐MS.

Several studies showed that rate constants for ^1^O_2_‐photoxygenation reactions on α‐NH_2_‐substituted histidines are in the same order of magnitude as for the free amino acid indicating that the major target for photooxidation is the imidazole group of histidine.^33^, ^36^ Notwithstanding this, many of the compositions that remained unchanged after irradiation, such as the Amadori rearrangement product (ARP) and other MRPs of the initial and intermediate phase (Figure 5), also contain intact histidine residues. Furthermore, when screening for imidazole fragments in MS/MS data, we could not observe a preferred degradation selectivity when MRPs contained intact imidazole groups (Figure S5, Supporting Information). The MS/MS spectra further revealed that most of the molecules formed upon irradiation still contained intact imidazole functions (Figure S5). Conclusively, imidazoles cannot be the dominating targets for photooxidation reactions but the nature of substituents at the α‐NH_2_ position seems to play a decisive role in the reactivity towards photons. It has been shown that the pH in aqueous solutions strongly affects the ^1^O_2_ oxygenation of histidine residues, indicating that oxygenation mainly occurs on unprotonated imidazole residues.^37^ The initial pH of the histidine model systems used in this study was equal to the p*K*_a_ value of the histidine side chain (pH 6). Within eight hours of irradiation, the pH value had dropped to about pH 5.5 (Tables S1–S2, Supporting Information). Consequently, the amount of unprotonated imidazoles in the model systems strongly decreased and it is conceivable that other moieties predominate in the photochemical degradation of histidine derived MRPs.

#### Photochemical selectivity

Interestingly, molecular formulae of the photochemically degraded species cover a discrete area in the van Krevelen space (Figure 4 c; Figure S2c and Figure S3c, Supporting Information). The majority of degraded compounds have small H/C and O/C ratios, which are characteristic of unsaturated and aromatic compounds.^28^ In all model systems, most of the photochemically degraded compounds were characterized by an O/C ratio ≤0.5 and an H/C ratio ≤1.5. Although the different amino acids led to the formation of very different, largely amino acid specific MRPs (Figure S6), this “photolabile area” was the same for all model systems. In the photolabile area, we reproducibly found 617, 611, and 851 molecular formulae, accounting for 34, 21, and 57 % of the total peak intensity, in the ribose–lysine, –arginine, and –histidine model systems, respectively. This somewhat higher number and higher molar amounts (estimated as relative peak intensities) found in the ribose–histidine MR may explain to a certain extent the higher number of degraded MRPs found in the histidine MR.

Several studies reported significantly greater antioxidant activity of MRPs formed from histidine compared to other amino acids.^38^ Indeed, antioxidants exhibit compositional characteristics (H/C and O/C ratios; Figure S7, Supporting Information) similar to the compounds found in the photolabile area shown in Figure 4. Hence, increased active oxygen and radical scavenging activity^39^ found for these MRPs may also play a role in the photochemical selectivity and the greater number of photomodified MRPs in the histidine model system.

**Figure 6:**
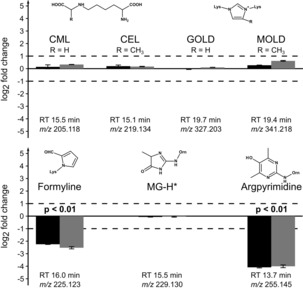
LC‐MS/MS analysis of known AGEs and MRPs that can be formed in the ribose–lysine and –arginine Maillard reaction, respectively. Log_2_ fold changes represent the changes in peak intensities between irradiated (8 h) and unirradiated control samples. Two independent irradiation experiments (experiment A: dark grey, experiment B: grey) were carried out. Each experiment was analyzed in triplicate injections by LC‐MS/MS.

Only a few AGE markers, which can be formed in the ribose–lysine and –arginine MR, have been described previously.^40^ We could identify seven compounds in our LC‐MS/MS data (Figure 6), and we used fragmentation spectra to substantiate chemical structures (Figures S8–S9, Supporting Information). Among the seven identified candidates, only formyline^41^ and argpyrimidine^24^ showed significant degradation (log_2_FC<−1 and p<0.01, Student's t‐Test (*n*=3)) after an irradiation time of eight hours in both irradiation experiments. Heterocyclic aromatic groups (pyrrole and pyrimidine) characterize these markers. In the two imidazole derivatives GOLD and MOLD^42^ both nitrogen atoms are substituted leading to a positive charge and therefore a reduced electron density in the aromatic ring structure, similar to protonated histidine residues. This indicates a selectivity of photochemical degradation reactions towards the degradation of electron‐rich aromatic heterocycles, which are preferentially formed in the final phase of the MR and often responsible for the characteristic browning (melanoidins).^1^, ^43^

#### Photoinduced formation of MRPs

In all model systems, we found far more compounds, which were produced upon simulated solar irradiation than MRPs that were degraded. More precisely, analysis of the ribose–histidine model system showed 1178 compounds after irradiation, which significantly increased in their peak intensities (log_2_FC >1, *p*<0.01, Student's t‐Test (*n*=3)) or were newly formed (Figure 4). By comparison, the ribose–lysine and arginine model systems showed 167 and 525 elemental compositions increasing in intensity after an irradiation time of eight hours, respectively (Figures S2–S3, Supporting Information). Compared with the degraded MRPs, the photochemically formed products showed a clear shift towards higher O/C ratios indicating that oxidation reactions are involved in the photochemical modification of MRPs. Although most of the photooxidation products can be formed by successive oxidation of double bonds (O/C>0.5 and H/C≤1.5), we also found a considerable number of photoproducts with an H/C ratio greater than 1.5 (Figure 4 c). These reaction products might be formed by disproportionation reactions or by reactions on carbonyl groups (e.g. hydroxy‐carbonyls or deoxyosones) with nucleophilic components of the system. Many of the degraded MRPs in the photolabile area may also serve as good scavengers for (hydroxy)‐acyl radicals formed by Norrish‐type reactions during the irradiation process.^14^ In general, these type of photoproducts have similar elemental compositions as thermally formed MRPs found in the initial and intermediate phase of the MR (Figure 5).

**Figure 7:**
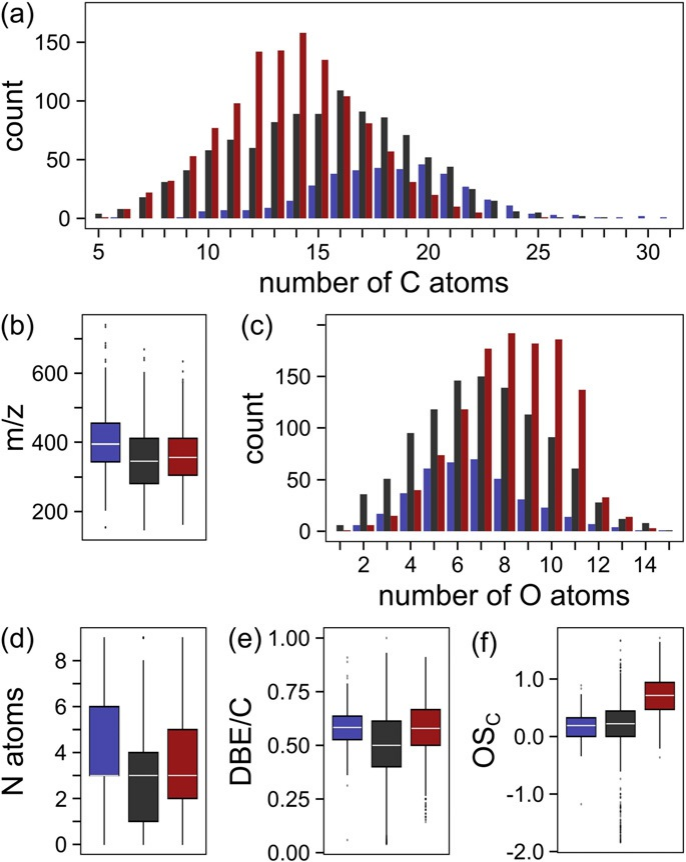
Overview of compositional descriptors retrieved for the ribose–histidine model system after molecular‐formulae computation from FT‐ICR‐MS data. Bar charts are grouped into features, which showed a significant decrease (blue; log_2_FC<−1 and p<0.01, Student's t‐Test (*n*=3)) and significant increase (red; log_2_FC >1 and p<0.01, Student's t‐Test (*n*=3)) in peak intensities in both independent irradiation experiments, respectively. Features that did not show a significant change in peak intensities after an irradiation time of eight hours are colored in black. Represented descriptors are (a) number of carbon atoms per formula, (b) measured *m*/*z*‐values, (c) number of oxygen atoms per formula, (d) number of nitrogen atoms per formula, (e) number of double bond equivalents per carbon atom, and (f) average carbon oxidation state.

We further studied the behavior of different compositional descriptors, which can be retrieved from the computed molecular formulae (Figure 7 and Figures S10–S11, Supporting Information). Interestingly, all model systems showed similar behavior, which can be summarized as follows:

(i) MRPs with a higher molecular weight and a larger number of carbon atoms preferably undergo photolytic reactions. By comparison, photochemical products tend to be smaller molecules with a reduced number of carbon atoms (Figure 7 a), indicating that cleavage mechanisms are likely to be involved in photochemical reactions on MRPs.

(ii) Upon photolytic reactions, the number of oxygen atoms per molecule increased on average by 1.5 oxygen atoms per molecule (Figure 7 c). Together with the increase in the average carbon oxidation state (Figure 7 f), it can be concluded that exogenous ROS, such as ^1^O_2_ or hydroxyl radicals, must play a key role in the photochemical reaction mechanisms.

(iii) Photosensitive MRPs have a higher number of double‐bond equivalents (sum of double bonds and rings) per carbon atom (DBE/C) than MRPs that are stable towards light exposure (Figure 7 e). However, we could not observe a noticeable difference between the DBE/C values of the degraded and the photochemically produced compounds. For example, after cleavage of a carbon–carbon double bond each of the two carbon atoms must still contain a double bond to maintain the DBE/C value. Given that the average carbon oxidation state of the photolysis products tends to have higher values than that of the other MRPs, it is likely that double bonds undergo photooxidative cleavage reactions leading to the formation of carbonyl moieties such as aldehydes or carboxylic acids. A decrease of the pH‐value during the irradiation experiments (Tables S1–S2, Supporting Information) further supports the formation of carboxylic acids in the course of photolysis.

(iv) Photolabile MRPs tend to be nitrogen‐rich compounds (Figure 7 d). When irradiated, the number of nitrogen atoms in the reaction products decreased. This substantiates photochemical targets, such as nitrogen‐containing heterocyclic structures or Schiff bases that might undergo oxidative degradation similar to the Karstens and Rossbach mechanism.^44^

#### Amino acid‐specific photochemical reactions and their orthogonality to thermal reactions

**Figure 8:**
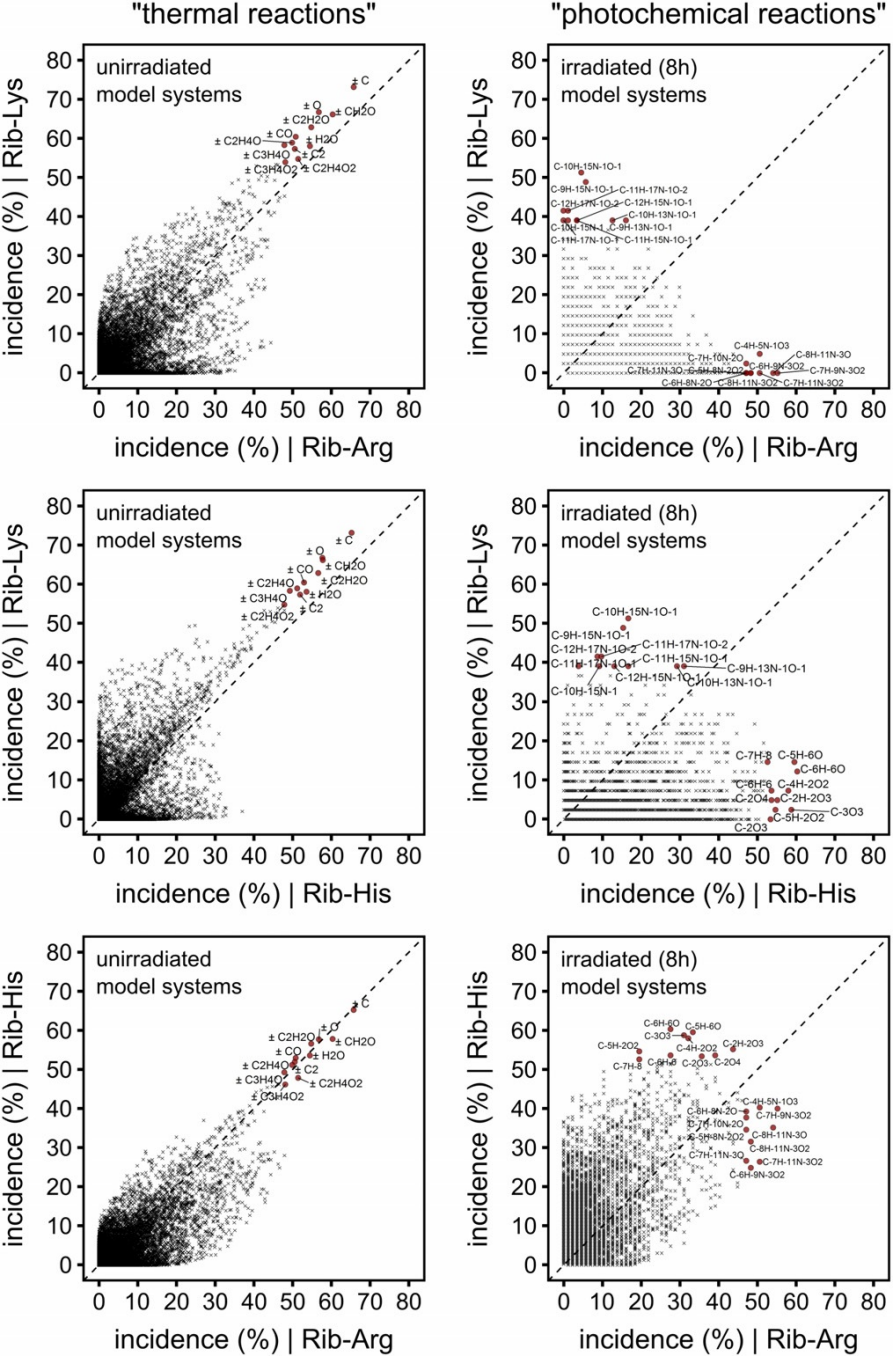
Pairwise comparison of mass difference incidences. Incidence rates were computed from all recorded mass differences in the mass spectra of thermally synthesized MRPs (left panel) and photochemically synthesized products (right panel). The top ten of the most frequently occurring mass differences were assigned to their element compositional equivalents representing possible net chemical transformations. Incidences represent the relative probability by which a monoisotopic signal in the mass spectrum can be linked to another monoisotopic signal with a given mass difference md_i_.

Nontargeted analysis aims to comprehensively investigate a sample's chemical composition. Although we are unlikely to be able to resolve and detect the entire chemistry of very complex samples, we can potentially find many precursor–product pairs in the mass spectra obtained from nontargeted experiments of reaction systems. The mass difference between a potential reaction precursor and product can provide information about their net chemical transformation.^45^ Although not all mass differences correspond to a real chemical transformation, they can provide useful information about the compositional connectivity between the observed reaction products.^46^ We computed all mass differences between all monoisotopic ions observed in the FT‐ICR mass spectra and used their relative incidences to elucidate meaningful mass differences (Figure 8). For example, an incidence rate of 54 %, as found for the mass difference 18.010565 Da (compositional equivalent: ±H_2_O) in the unirradiated ribose–histidine model system (Figure 8), means that 54 % of all monoisotopic ion signals in the spectra can be connected to another signal by this mass difference.

Pairwise comparison of the different unirradiated control model systems showed good correlation of mass differences, especially those with high incidence rates, indicating very similar chemical reactions in the thermal formation of MRPs. These mass differences involved chemical transformations, such as (de)hydration (±H_2_O) and oxidation/reduction (±O), which are known to play a crucial role in the thermal synthesis of MRPs. This data agrees with our recent study, which showed that different amino acid precursors follow consistent reactivity behavior, even though the different amino acid precursors lead to very different chemical compositions.^47^ In contrast, when comparing mass differences, which were exclusively found between degraded MRPs (“photoprecursors”) and compounds formed upon irradiation (“photoproducts”) we obtained a completely different picture (Figure 8). First, we observed poor correlation of reactivity patterns between the different model systems compared with the thermally formed MRPs, indicating that photochemical reactions of MRPs are very specific to the parent amino acids. Second, net chemical transformations that are dominating the MR in thermal processing seem to play only a minor role in the photochemical reactions of the MRPs. It is worth noting that intermediate reactions, which do not lead to stable molecules detectable by MS, are not considered with this approach. Conclusively, although major chemical reactions in the thermal synthesis of MRPs are very consistent and independent of the amino acid precursor, the same systems show strong amino acid specificity in the photochemical degradation of MRPs.

## Conclusions

We studied the effects of simulated solar radiation on the modification of MRPs, formed in a typical non‐enzymatic browning reaction at moderate temperature (100 °C). Upon photon absorption, hundreds of MRPs readily underwent degradation reactions leading to a complex mixture of newly formed photoproducts. Our data provides evidence for a strong selectivity of photodegradation reactions, mainly towards electron‐rich and nitrogen‐containing heterocycles, which are preferentially formed in the advanced and final phase of the Maillard reaction. Photoreactions on these structures break down the molecules into smaller but strongly oxidized compounds. Although the “traditional” (thermal) synthesis of amino acid glycation products follows general chemical reactions, such as dehydration, carbonyl cleavage, and redox reactions, photoreactions are a lot more diverse and show strong amino acid specificity. This fundamental study is of special importance in the shelf‐life of foods, phototoxicity mechanisms in diabetic and aged tissues and may, under certain conditions, also play a role in prebiotic molecular synthesis. Lack of current comprehensive database information on MRPs and photochemical products did not allow identification of structures, but also suggests a great pool of as yet unexplored chemical compounds, which need detailed characterization in future studies. Studies on purified reaction products may help to understand some specific photodegradation mechanisms. However, it must be taken into consideration that many of the formed photoproducts are likely to be produced only in a complex interplay of reactive intermediates and products. Hence, further improvements in holistic approaches are required to gain better understanding.

## Experimental Section

Chemicals and reagents l‐Arginine (≥98 %), l‐asparagine (>99 %), l‐histidine (98 %), l‐lysine (>98 %), and d‐(−)‐ribose (98 %) were purchased from Sigma–Aldrich (Steinheim, Germany). Urea (100 %) was obtained from Beckmann Coulter (Krefeld, Germany). LC‐MS grade methanol and acetonitrile were purchased from Merck (Darmstadt, Germany). Formic acid (LC‐MS grade) and ammonium formate (10 m stock solution) were obtained from Sigma–Aldrich (Steinheim, Germany). MilliQ‐purified water (18.2 MΩ; Millipore, Germany) was used throughout the experiments.

### Maillard model systems

Equimolar mixtures of ribose and amino acids (0.1 mol L^−1^ respectively) were prepared in MilliQ‐purified water. 1 mL of each mixture was heated in closed glass vials as recently described at 100 °C for ten hours.^27^ Model systems were stored at −20 °C until usage.

### Irradiation experiments

All Maillard model systems were diluted 1:800 (*v*/*v*) with MilliQ‐purified water prior to irradiation. Aliquots of the model systems were irradiated in a custom‐built photolysis system, as described in detail elsewhere.^48^ Irradiation experiments were performed for 20 h with a 1000 W Xe arc lamp equipped with an air mass filter (AM 1.5). Before each irradiation experiment, the lamp intensity was controlled to ensure that the irradiated sample receives a radiation dose, which is equivalent to the sun at Earth's surface (45° north, midsummer, at noon). The temperature was controlled at 25 °C using Peltier units and a circulating water bath. The pH value was monitored throughout the irradiation process (Table S1, Supporting Information).

For subsequent FT‐ICR‐MS and LC‐MS/MS analysis, larger sample amounts were irradiated, with the same dilution as above (1:800 (*v*/*v*)), in quartz vessels in a Suntest CPS system (Heraeus, Hanau, Germany) equipped with an NXE xenon lamp (Atlas Material Testing Technology, Gelnhausen, Germany) for four and eight hours. The temperature was maintained at 25 °C using an air conditioning unit and the pH was recorded before and after irradiation (Table S2), respectively. Additionally, control samples in lightproof vessels were placed under the xenon lamp. All samples were irradiated in two independent experiments (*n*=2).

### Excitation emission matrix fluorescence

Online EEM measurements during the irradiation were performed every 20 min using a 4×10 mm flow cell and an Aqualog spectrofluorometer (Horiba Instruments, New Jersey, USA). Excitation ranged from 230 to 600 nm and emission was recorded between 211–617 nm. All fluorescence spectra were corrected for scatter and inner filter effects. Normalization to a 1 mg L^−1^ quinine sulfate standard (Starna reference material RM‐QS00, 1.28×10^−6^ mol L^−1^) was used to express all fluorescence intensities in quinine sulfate units (ppm). Independently for each irradiated model system, PARAFAC models were built using the drEEM toolbox for MATLAB.^25^ The data best fitted four‐component PARAFAC models, which were split‐half validated, explaining 99.8, 99.8, and 99.7 % of the spectral variance in the ribose–lysine, –arginine, and –histidine model system, respectively.

### FT‐ICR‐MS analysis

All irradiated Maillard model systems were further diluted with methanol to achieve a final dilution of 1:2500 (*v*/*v*) immediately prior to FT‐ICR‐MS analysis. Each sample was analyzed in three independent injections (*n*=2×3=6 MS measurements). Direct‐infusion FT‐ICR mass spectra were acquired with a 12 T Bruker Solarix mass spectrometer (Bruker Daltonics, Bremen, Germany). Samples were infused with a flow rate of 2 μL min^−1^ into an APOLLO II electrospray source operated in negative ionization mode. Ion‐source settings and spectra calibration were the same as recently described.^27^ Spectra were acquired with a time‐domain of 4 megawords and 300 scans were accumulated within a mass range of *m*/*z* = 123–1000.

Peaks with a signal‐to‐noise ratio of at least eight were exported to mass lists. Data prefiltering was used to remove FT artifacts,^49^ features with unusual mass defects and ^13^C isotope signals. Peaks were then aligned into a matrix containing averaged *m*/*z*‐values and corresponding peak intensities allowing a maximum alignment window of 1 ppm.^50^ Only those *m*/*z*‐values were retained in the matrix, which were reproducibly found in all three replicate injections of at least one sample. Zero values in the matrix then were replaced by the recorded absolute intensity values found in the raw spectra at that *m*/*z*‐value, respectively. Finally, molecular formulae were computed for all averaged *m*/*z*‐values as recently described.^47^

### Tandem HILIC‐RP LC‐MS/MS

Aliquots (16 mL) of the irradiated samples were lyophilized until dryness and immediately reconstituted in 200 μL of an aqueous solution containing 2 % acetonitrile prior to LC‐MS/MS analysis. Instrumental setup and chromatographic conditions were the same as recently described.^26^ Each sample was injected and analyzed in triplicate. The MS data were recorded with a high‐resolution Bruker maXis qTOF‐MS equipped with an APOLLO II electrospray ion source (Bruker Daltonics, Bremen, Germany), which was operated in electrospray positive mode to achieve maximum compound coverage.^26^ Precursor and product ion scans were recorded in a mass range from *m*/*z*=50–1500 with a scanning rate of 5 Hz. For data‐dependent fragmentation, after each precursor scan, the two most abundant precursors were isolated and subjected to collision‐induced fragmentation. Maximum coverage of MS/MS data was achieved by excluding precursor masses from fragmentation after three successful MS/MS spectra for five minutes. The collision energy was set to 35 eV. All mass spectra were internally calibrated by infusing a tuning mix solution (Agilent Technologies, Waldbronn, Germany) prior to each chromatographic run.

Raw data were post‐processed using the XCMS R package (version 3.2.0).^51^ Chromatographic features were detected by the *centWave* algorithm^52^ using an expected approximate peak width in the range from 10–80 s and a maximum tolerated *m*/*z* deviation of 10 ppm. Retention time alignment was done with the *Orbiwarp* algorithm as integrated in XCMS.^53^ Peaks within and between samples then were grouped into chromatographic features (retention time‐*m*/*z*‐pairs) based on time dimension densities.^51^ In the obtained matrix, only those features were retained, which were reproducibly detected in all three replicate injections of at least one sample.

### Data analysis

All further statistical analysis and filtering was done in R Statistical Language and Microsoft Excel 2016. All *p*‐values were calculated based on heteroscedastic Student's t‐Tests. The number of double‐bond equivalents per carbon atom (DBE/C) and average carbon oxidation state (OS_C_) was computed as recently described.^27^, ^47^

## Conflict of interest

The authors declare no conflict of interest.

## Supporting information

As a service to our authors and readers, this journal provides supporting information supplied by the authors. Such materials are peer reviewed and may be re‐organized for online delivery, but are not copy‐edited or typeset. Technical support issues arising from supporting information (other than missing files) should be addressed to the authors.

Supplementary
