# Supplementary material for: Simulated Sunlight Selectively Modifies Maillard Reaction Products in a Wide Array of Chemical Reactions
Source: Chemistry. 2019 Sep 13;25(57):13208–17. doi: 10.1002/chem.201902804 (PMC6856810; doi:10.1002/chem.201902804)
Supplement: Supplementary file 1 — Supplementary [file CHEM-25-13208-s001.pdf]

CHEMISTRY
A European Journal

Supporting Information

Simulated Sunlight Selectively Modifies Maillard Reaction Products in a Wide Array of Chemical Reactions

Daniel Hemmler,*[a, b] Michael Gonsior,[c] Leanne C. Powers,[c] James W. Marshall,[d] Michael Rychlik,[a] Andrew J. Taylor,[d] and Philippe Schmitt-Kopplin*[a, b, c]

chem_201902804_sm_miscellaneous_information.pdf

Holistic characterization of photosensitive MRPs

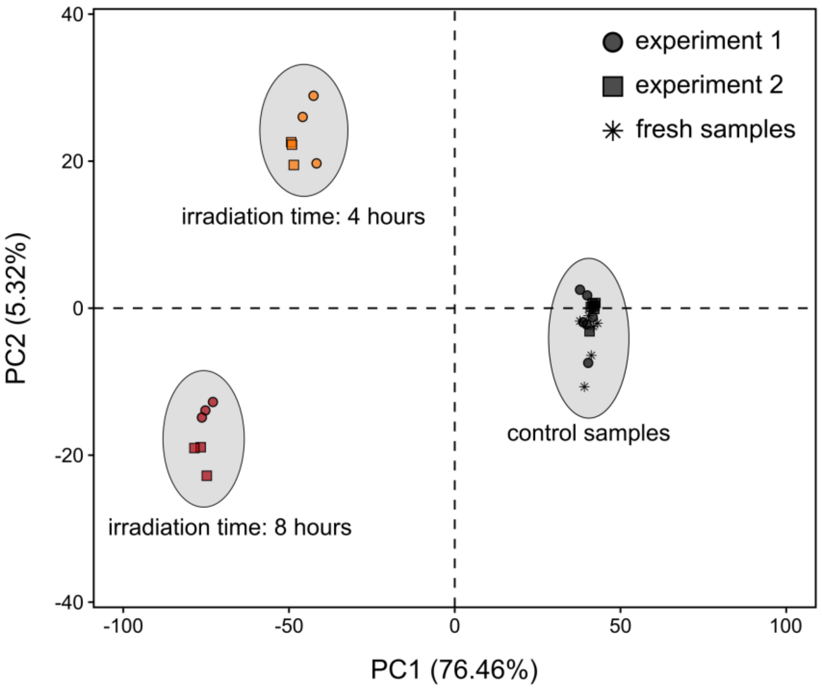

Figure S1 Principal component analysis of ribose-histidine FT-ICR-MS raw data. Samples were irradiated for four and eight hours in a suntester system. Additionally, control samples, which were kept under the same conditions, however, protected from light exposure, as well as freshly prepared model systems were analyzed. All experiments were carried out in two independent experiments. Each sample was further injected in triplicate measurements (total number of samples per treatment = 6). Samples and replicate injections were measured in randomized order.

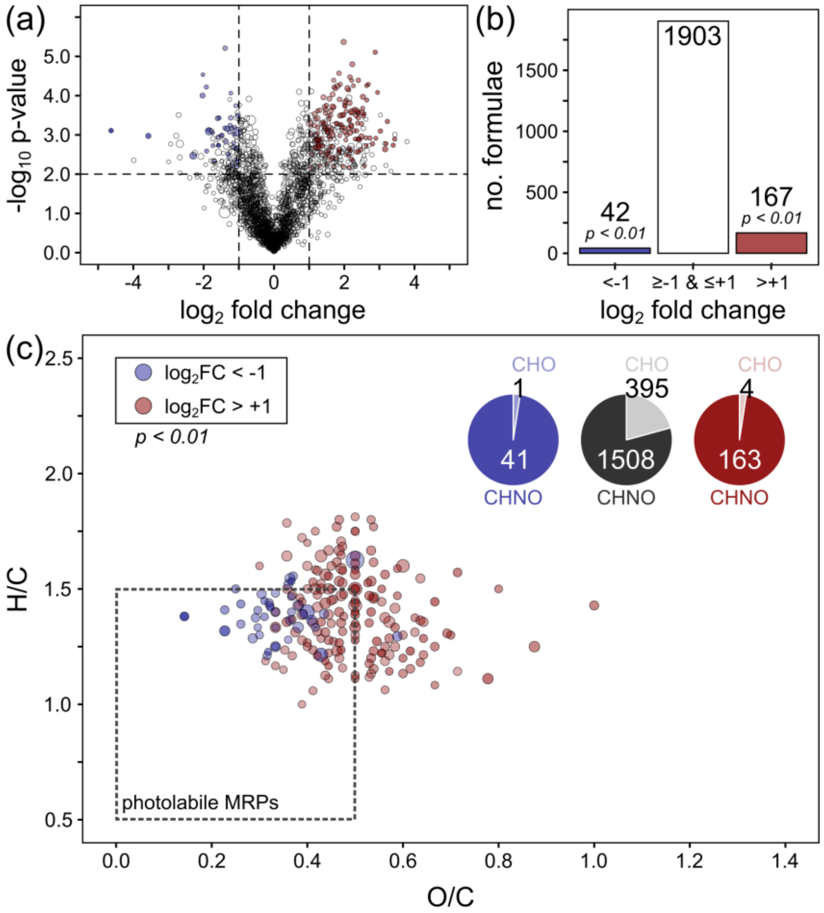

Figure S2 Effect of solar irradiation on elemental compositions of **ribose-lysine MRPs**. Model systems were irradiated for eight hours and compared to unirradiated control samples. Irradiation experiments were performed in duplicate. Each sample then was analyzed by FT-ICR-MS in triplicate injections (N = 2 × 3). Peak intensities of all features found in irradiated samples were compared to the same features in the unirradiated control samples by Student's t-Test (n = 3): Features, which showed a significant decrease in peak intensities in both independent irradiation experiments are colored in blue. Features, which showed a significant increase or were newly formed upon irradiation are highlighted in red, respectively. (a) Volcano plot. (b) Number of molecular formulae showing significant changes in peak intensities. (c) Van Krevelen diagram of all significantly affected molecular formulae. Pie charts illustrate the reduced occurrence of nitrogen-free (CHO) MRPs in photochemical reactions. Black pie chart represents elemental compositions, which did not show a significant change in peak intensities upon irradiation.

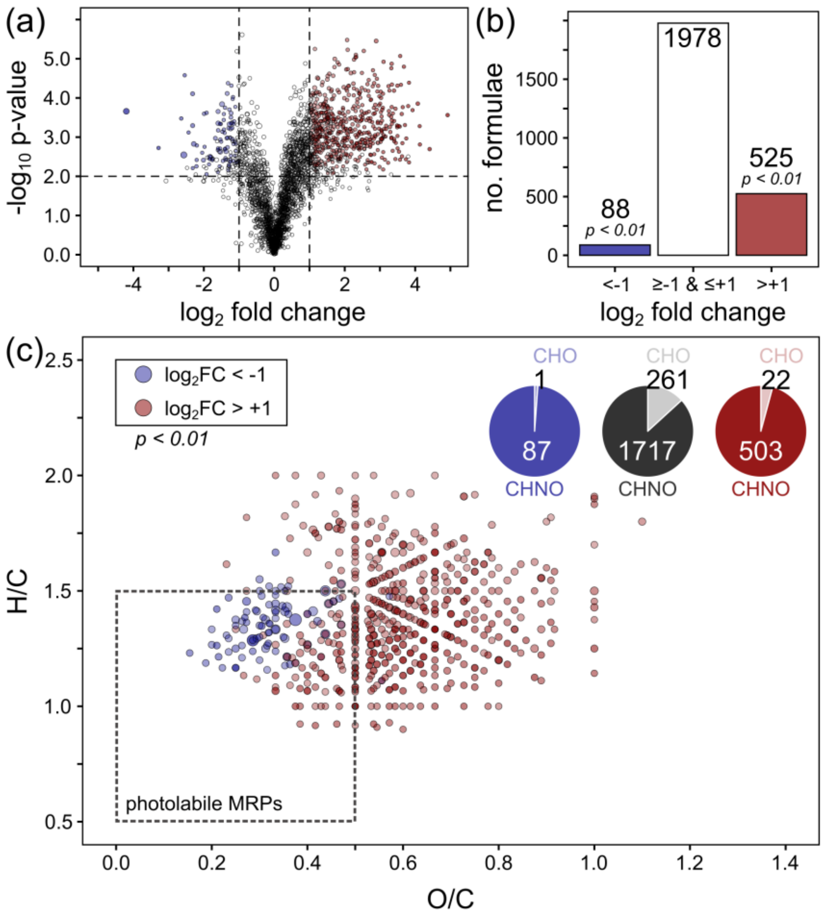

Figure S3 Effect of solar irradiation on elemental compositions of **ribose-arginine MRPs**. Model systems were irradiated for eight hours and compared to unirradiated control samples. Irradiation experiments were performed in duplicate. Each sample then was analyzed by FT-ICR-MS in triplicate injections (N = 2 × 3). Peak intensities of all features found in irradiated samples were compared to the same features in the unirradiated control samples by Student's t-Test (n = 3): Features, which showed a significant decrease in peak intensities in both independent irradiation experiments are colored in blue. Features, which showed a significant increase or were newly formed upon irradiation are highlighted in red, respectively. (a) Volcano plot. (b) Number of molecular formulae showing significant changes in peak intensities. (c) Van Krevelen diagram of all significantly affected molecular formulae. Pie charts illustrate the reduced occurrence of nitrogen-free (CHO) MRPs in photochemical reactions. Black pie chart represents elemental compositions, which did not show a significant change in peak intensities upon irradiation.

Photooxidation of MRPs by singlet oxygen

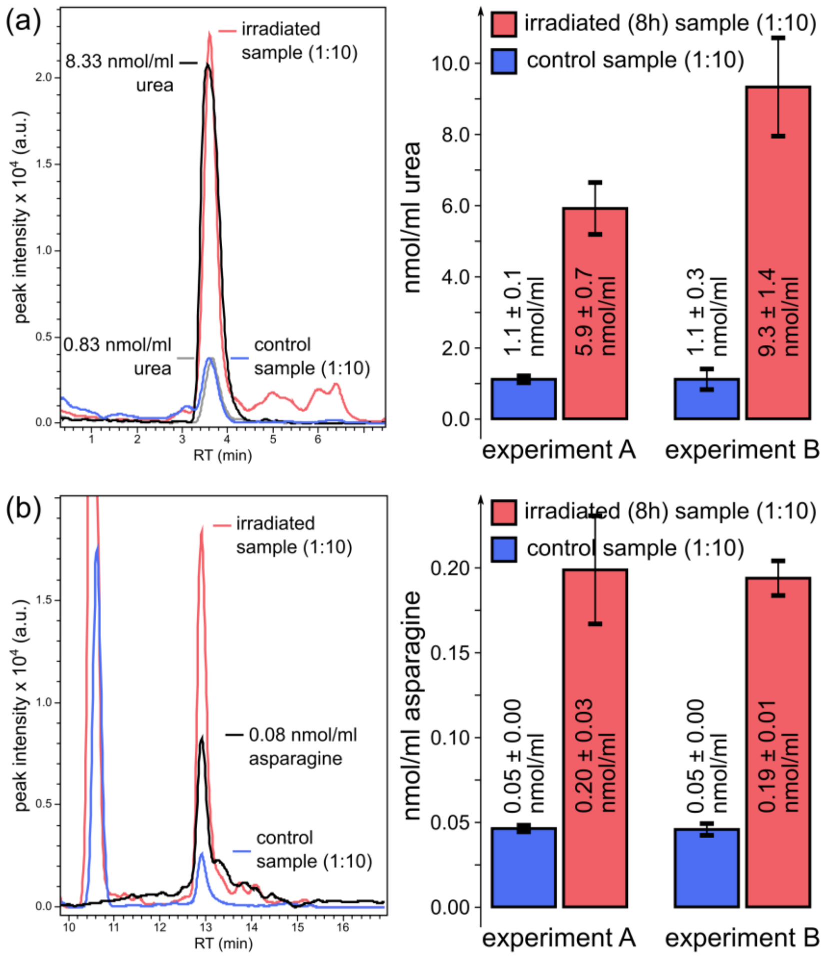

Figure S4 Quantification of (a) urea and (b) asparagine in ribose-histidine model systems. After lyophilization, the model systems were reconstituted in 2% acetonitrile solution to achieve a dilution factor of 1:10 (*v/v*) with respect to the original model system. Calibration curves were computed from analyzed standard solutions as shown below.

Calibration standards used for quantification of urea and asparagine in ribose-histidine model systems. Standards were prepared in 2% acetonitrile solution. Concentration values are given in nmol ml^{-1}.

calibrant	urea	L-asparagine
1	0.08	0.04
2	0.17	0.08
3	0.83	0.38
4	1.67	0.76
5	8.33	3.78
6	16.65	

The role of imidazole groups in photochemical reactions

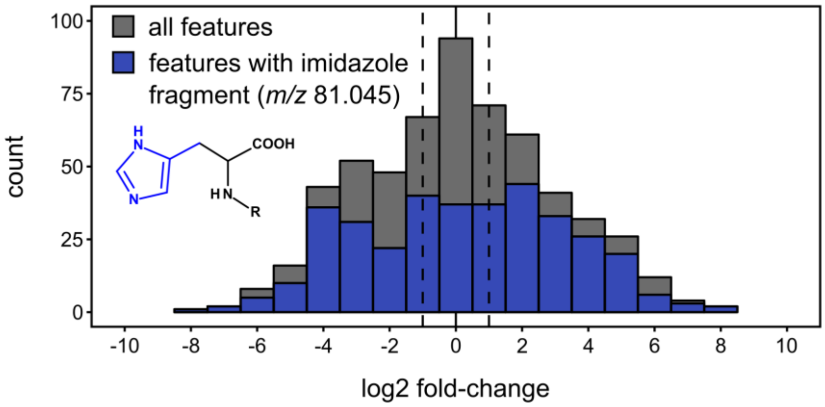

Figure S5 Screening of fragment spectra containing imidazole fragments at *m/z* = 81.045. Fragment spectra were acquired by data-dependent LC-MS/MS. Histograms illustrate the log_2 fold change (irradiated samples vs. control samples) of the peak intensities as influenced by irradiating ribose-histidine samples for eight hours. Grey histogram represents all chromatographic features with available MS/MS spectra (N = 580). Blue histogram shows only features, which contained an imidazole characteristic fragment (*m/z* = 81.045) in their MS/MS spectra (N = 355).

Changes in pH during irradiation experiments

Table S1. Measured pH values during irradiation of Maillard model systems in the custom-built photolysis system used for online EEM measurements.

Irradiation time	pH ribose-lysine	pH ribose-arginine	pH ribose-histidine
0 h (before irradiation)	6.06	6.64	6.01
4 h	5.48	6.29	5.64
8 h	5.30	6.14	5.48
12 h	5.19	6.10	5.42
16 h	5.14	6.07	5.38
20 h	5.10	6.07	5.37

Table S2. Measured pH values (mean from n = 2 irradiation experiments) before and after irradiation of Maillard model systems in the Suntest CPS system.

Irradiation time	pH ribose-lysine	pH ribose-arginine	pH ribose-histidine
0 h (before irradiation)	6.0	6.6	6.0
8 h (after irradiation)	5.2	6.1	5.4

Chemical diversity of thermally formed MRPs

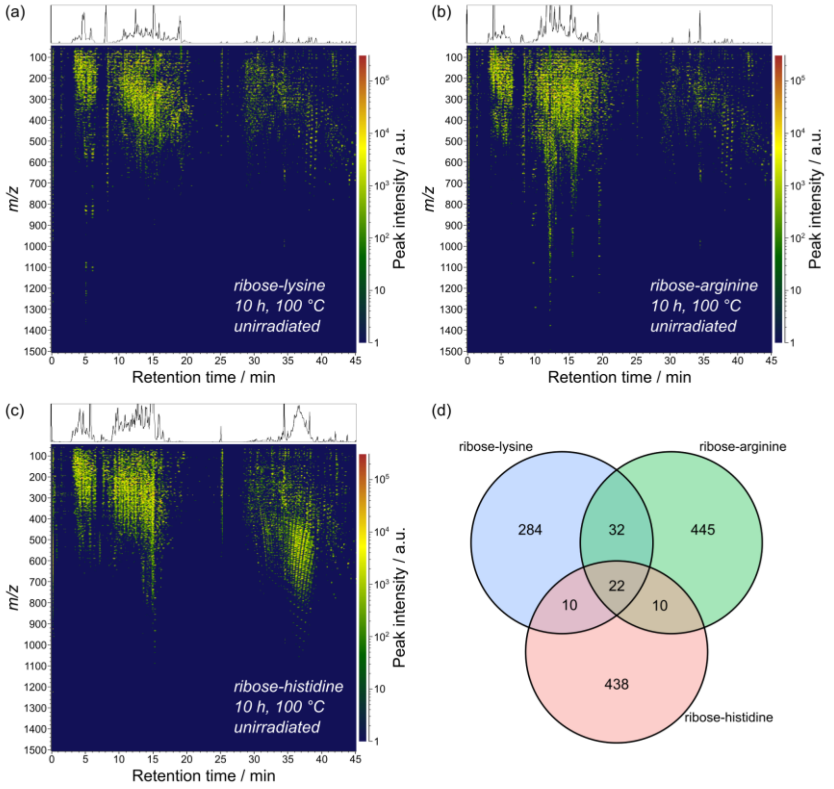

Figure S6 Non-target analysis of unirradiated model systems by tandem HILIC-RP chromatography. Retention time versus *m/z*-value plots of a (a) ribose-lysine, (b) ribose-arginine, and (c) ribose-histidine model system, heated for ten hours at 100 °C. Each dot in (a-c) represents an analytical signal (feature) colored according to the observed peak intensity. (d) Venn diagram illustrates the amino acid specific chemical diversity in produced MRPs. Between the three model systems, chromatographic features were considered as the same chemical compound when *m/z*-values and retention times were equal (*m/z*-alignment: ± 10 ppm and ΔRT ≤ 30 s).

Compositional description of antioxidants

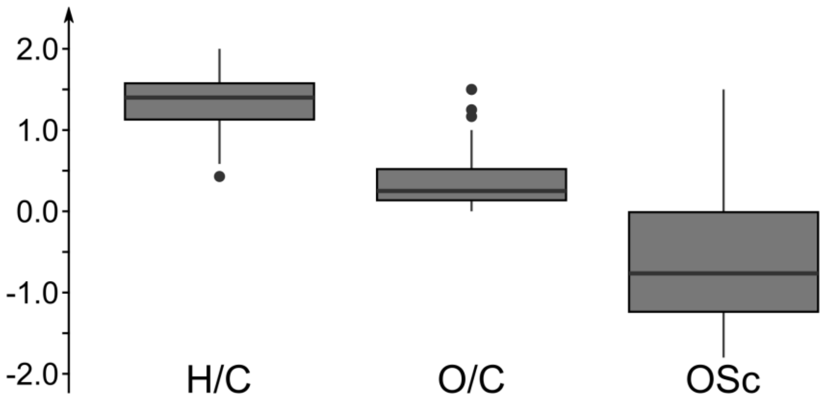

Figure S7 Compositional descriptors retrieved for 125 antioxidants containing no more than C, H, N, and O elements. Antioxidants were taken from FooDB (Release June 29, 2017).

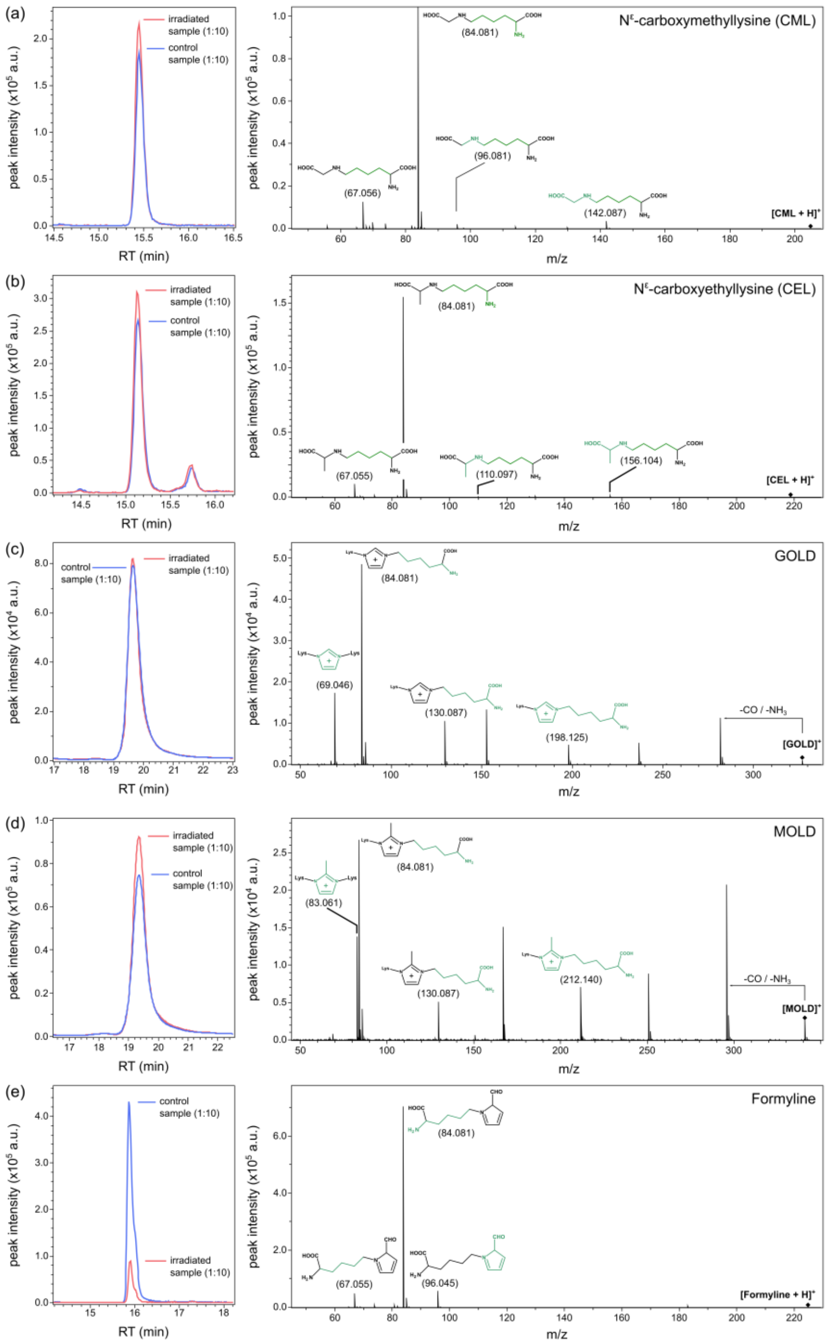

Figure S8 LC-MS/MS analysis of AGE markers in unirradiated ribose-lysine model systems (blue) and ribose-lysine model systems irradiated for eight hours (red). Structures were confirmed by MS/MS spectra. Analyzed markers were: (a) carboxymethyllysine (CML), (b) carboxyethyllysine (CEL), (c) GOLD, (d) MOLD, and (e) formyline.

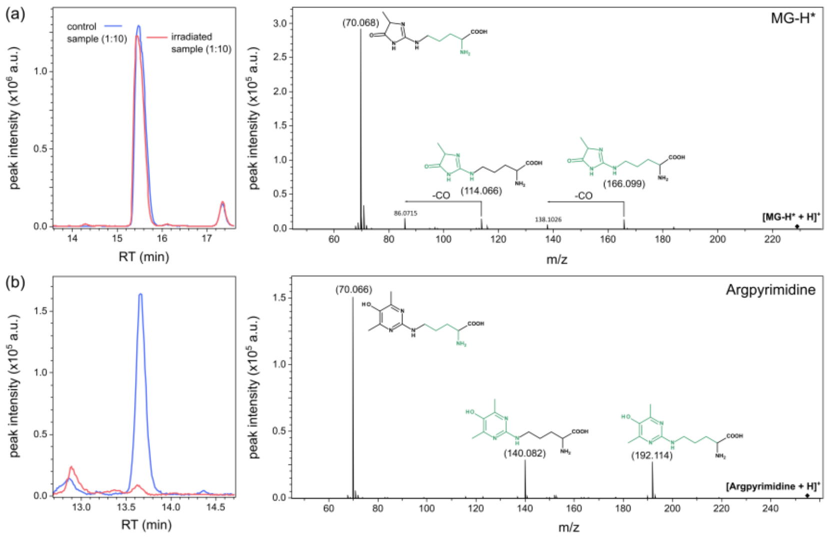

Figure S9 LC-MS/MS analysis of AGE markers in unirradiated ribose-arginine model systems (blue) and ribose-arginine model systems irradiated for eight hours (red). Structures were confirmed by MS/MS spectra. Analyzed markers were: (a) one hydroimidazolone isomer MG-H* (assignment of the three possible isomers MG-H1 – MG-H3 based on MS/MS data not possible), and (b) argpyrimidine.

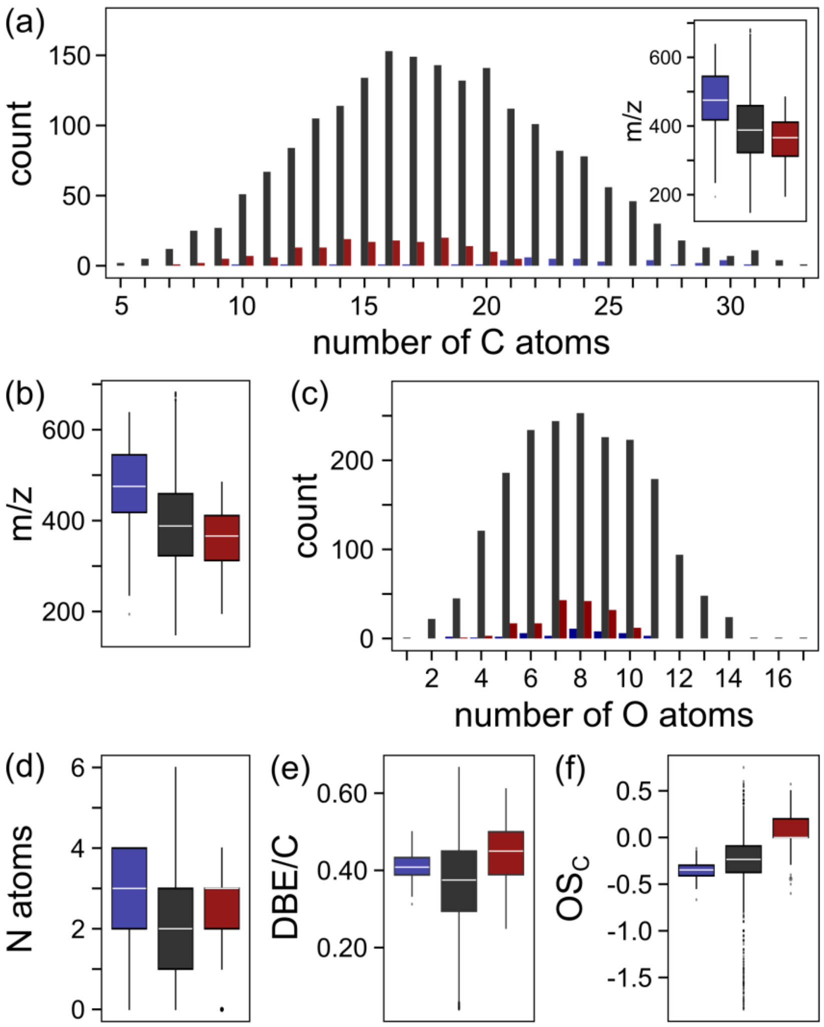

Figure S10 Overview of compositional descriptors retrieved for the **ribose-lysine** model system after molecular formulae computation from FT-ICR-MS data. Bar charts are grouped into features, which showed a significant decrease (blue; log2FC < -1 and p < 0.01, Student's t-Test (n = 3)) and significant increase (red; log2FC > 1 and p < 0.01, Student's t-Test (n = 3)) in peak intensities in both independent irradiation experiments, respectively. Features that did not show a significant change in peak intensities after an irradiation time of eight hours are colored in black. Represented descriptors are (a) number of carbon atoms per formula, (b) measured *m/z*-values, (c) number of oxygen atoms per formula, (d) number of nitrogen atoms per formula, (e) number of double bond equivalents per carbon atom, and (f) average carbon oxidation state.

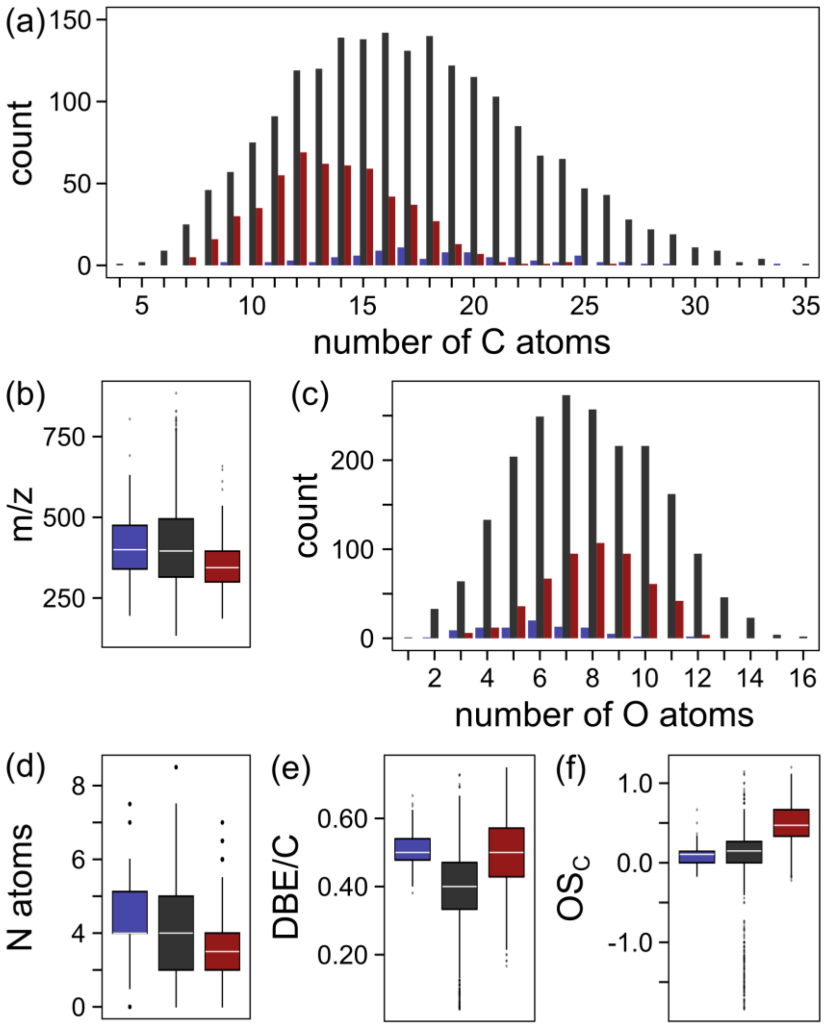

Figure S11 Overview of compositional descriptors retrieved for the **ribose-arginine** model system after molecular formulae computation from FT-ICR-MS data. Bar charts are grouped into features, which showed a significant decrease (blue; log2FC < -1 and p < 0.01, Student's t-Test (n = 3)) and significant increase (red; log2FC > 1 and p < 0.01, Student's t-Test (n = 3)) in peak intensities in both independent irradiation experiments, respectively. Features that did not show a significant change in peak intensities after an irradiation time of eight hours are colored in black. Represented descriptors are (a) number of carbon atoms per formula, (b) measured *m/z*-values, (c) number of oxygen atoms per formula, (d) number of nitrogen atoms per formula, (e) number of double bond equivalents per carbon atom, and (f) average carbon oxidation state.
